# Supplementary material for: Plasmodium sporozoite search strategy to locate hotspots of blood vessel invasion
Source: Nat Commun. 2023 May 23;14:2965. doi: 10.1038/s41467-023-38706-z (PMC10205706; doi:10.1038/s41467-023-38706-z)
Supplement: Supplementary file 1 — Supplementary Information [file 41467_2023_38706_MOESM1_ESM.pdf]

SUPPLEMENTARY INFORMATION

Plasmodium sporozoite search strategy to locate hotspots of blood vessel invasion

Pauline Formaglio[1]†, Marina E. Wosniack[2]†, Raphael M. Tromer[3], Jaderson G. Polli[4], Yuri B. Matos[4], Hang Zhong[1], Ernesto P. Raposo[5], Marcos G. E. da Luz[4]*, Rogerio Amino[1]*

[1] Institut Pasteur, Université Paris Cité, Malaria Infection and Immunity Unit; 75015, Paris, France.

[2] Max Planck Institute for Brain Research; 60438, Frankfurt, Germany.

[3] Departamento de Física Teórica e Experimental, Universidade Federal do Rio Grande do Norte; 59078- 970, Natal-RN, Brazil.

[4] Departamento de Física, Universidade Federal do Paraná; 81531-980, Curitiba-PR, Brazil.

[5] Laboratório de Física Teórica e Computacional, Departamento de Física, Universidade Federal de Pernambuco; 50670-901 Recife-PE, Brazil.

† These authors contributed equally to this work

*Corresponding authors. Email: roti@pasteur.fr, luz@fisica.ufpr.br

Supplementary Note 1. Velocity autocorrelation analysis

Considering the sporozoite velocities in the time instants t and t-1, with $\theta_{t,t-1}$ denoting their relative angle, we define the component

$$v_c(t) = |v(t)| \cos[\theta_{t,t-1}]$$ (Supplementary Equation 1).

Then, we can calculate the velocity autocorrelation function of lag τ as [1] (for T the maximum time):

$$c(\tau) = \frac{\sum_{t=0}^{t=T-\tau}(v_c(t)-\langle v_c\rangle)\,(v_c(t+\tau)-\langle v_c\rangle)}{\sum_{t=0}^{t=T}(v_c(t)-\langle v_c\rangle)^2}$$ (Supplementary Equation 2)

The results for INV and NINV, both in the states St1 and St2, are shown in the Supplementary Figure 1. These graphs have a temporal scale limitation due to the data processing method. Successive times t and t-1 represent, in fact, averages over intervals of four seconds, establishing a lower-bound for the temporal resolution. Also, autocorrelation statistics tend to be very poor for rather short temporal series, here corresponding to individuals with less than 30 steps (120 seconds) for INV and with less than 100 steps (400 seconds) for NINV. These samples were therefore eliminated from the analysis. All this, of course, hinders a high precision estimation for the auto-correlation rate decay. Furthermore, for the St1 state, the velocities and step lengths tend to be considerably smaller, conceivably making C(τ) to be more susceptible to other factors, like the dermis local structures. Even then, expected general trends can be observed in the Supplementary Figure 1. First, in all cases, after 4 s (the minimum acquisition time) the correlation decay is slow and positive up to a certain τmax. Second, positive autocorrelation during a certain time characterizes a specific movement mode: very small steps and low speed for mode St1 and larger steps and higher speeds for mode St2. For τmax = 20 s for INV and τmax = 40 s for NINV, the autocorrelation is positive and higher for St2 than for St1. For the state St2 (specially for the NINV) such τmax fairly corresponds to the threshold for which the Hurst exponent is higher than 0.5 (Fig. 3a).

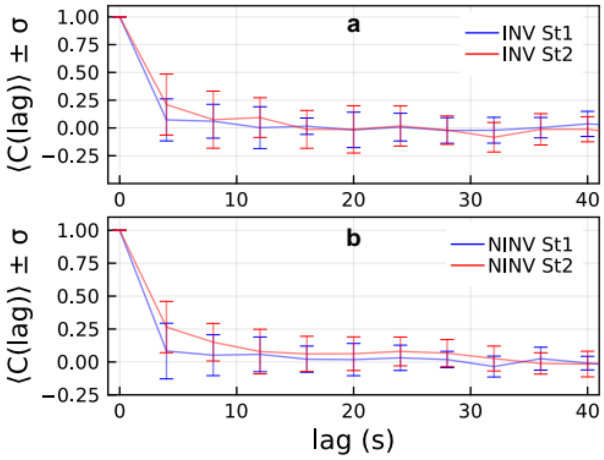

Supplementary Figure 1. Velocity autocorrelation. Velocity autocorrelation (supplementary equation 2) for the state 1 (St1, blue) and state 2 (St2, red) modes of (a) invader (INV) and (b) non-invader (NINV) parasites for the lag, i.e. τ, given in seconds. Lines represent the average and whiskers the standard deviation. Pooled data from 7 independent experiments (n=188 tracks) yielding 10 analyzable segments for St1 INV, 15 for St2 INV, 16 for St1 NINV and 20 for St2 NINV.

Supplementary Note 2. Lévy walks and Lévy flights

Lévy walks and flights have been successfully applied to model a wide variety of phenomena displaying super-diffusive dynamics in many fields [2, 3]. Sometimes these jargons are misleadingly used in the literature as meaning the same kind of process. In fact, although similar in some points, Lévy walks and flights differ in a number of important features. Lévy random walkers and fliers present step lengths drawn from the family of Lévy α-stable distributions [2, 3], with the Lévy index in the range $\alpha \in (0, 2]$ (the limit case $\alpha = 2$ corresponds to the Gaussian distribution).

While Lévy flights are Markovian processes with jump duration (generally instantaneous) independent of the length and diverging second moment for $\alpha \in (0, 2)$, steps in Lévy walks are taken with finite (generally constant) speed, thus generating spatio-temporal correlations leading to non-Markovian temporal evolution and converging second moment [4, 5, 6, 7, 8]. As closed mathematical expressions for Lévy α-stable distributions in terms of simple functions are known only for a few cases [2, 3] (e.g., the non-skewed Cauchy distribution with $\alpha = 1$), in many instances power-law distributions of step lengths ℓ in the form $P(\ell) = C/\ell^{\mu}$, where $\ell \geq \ell_{min}$ and $\mu = \alpha + 1$ in the range $1 < \mu < 3$ (with ℓ_{min} a lower cutoff length and C a proper normalization constant [1]), have been applied to describe super-diffusive dynamics, since $P(\ell)$ corresponds to the asymptotic large-ℓ limit of Lévy distributions with $0 < \alpha < 2$ [2, 3]. We note that decreasing μ values within the interval $1 < \mu < 3$ translate into an increasing degree of super-diffusiveness, with the limit $\mu \to 1$ (i.e., $\alpha \to 0$) leading to ballistic behavior consisting essentially of extremely large steps [1]. The case $\mu = 3$ ($\alpha = 2$) corresponds [1] to normal diffusion typical of Brownian-like motion (power-law distributions with exponent $\mu > 3$ also display normal dynamics due to the central limit theorem).

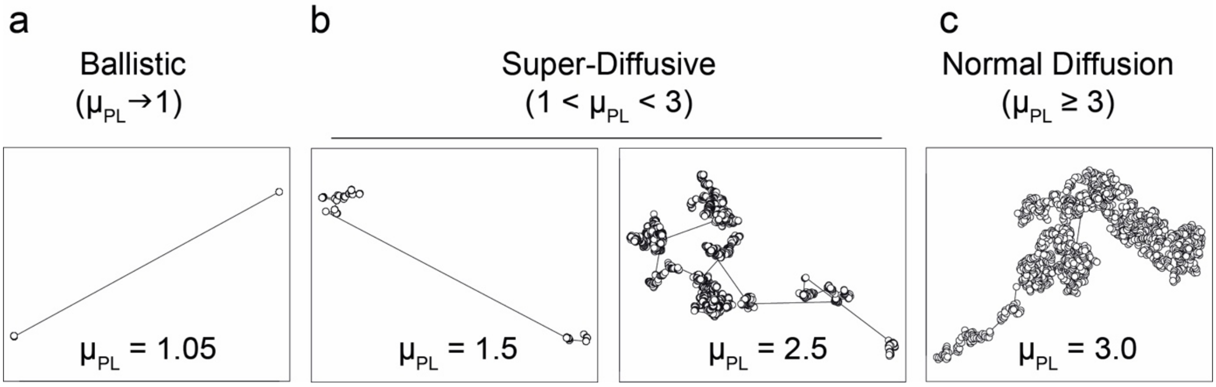

Supplementary Figure 2. Relationship between diffusive behavior and μ_{PL}. Lévy walk simulations [9] illustrating μ_{PL} (bottom of each simulation) and its relationship with (**a**) ballistic behavior (limit $\mu_{PL} \rightarrow 1$) , (**b**) super-diffusive behaviors ($1 < \mu_{PL} < 3$), and (**c**) normal diffusion ($\mu_{PL} \geq 3.0$). Panels highlight how smaller μ values lead to larger steps and enhanced diffusion, while for larger μ values (μ=3), smaller steps dominate the motion leading to normal diffusion. Graphs were created with the adehabitatHR package of the R software.

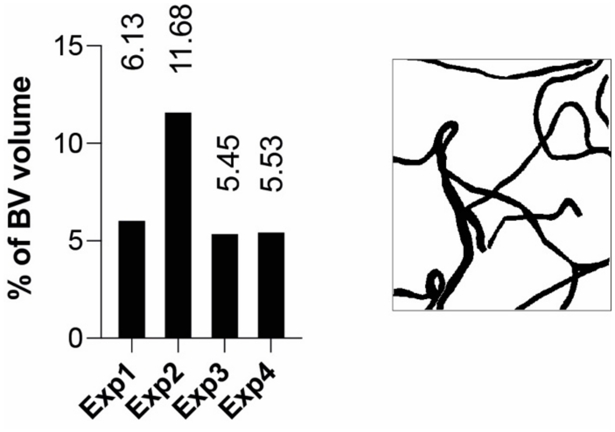

Supplementary Figure 3. Fractional volume of blood microvasculature in mouse skin. Blood vessel (BV) outlines and skeleton were extracted from intravital imaging data as described in the Methods section. To calculate the volume occupied by the microvasculature within the imaged volume, the length of each branch of the BV skeleton (L_{BRANCH}) was next measured using the Lines8 plug-in. The diameter of each branch (D_{BRANCH}) was then determined by (i) measuring the diameter on the plane where the BV is the largest (using maximum intensity projection) and (ii) averaging values from measurements performed at regular intervals along the branch. The volume of each branch (V_{BRANCH}) was estimated by considering BV as cylinders and calculated as: $V_{BRANCH} = \pi*(D_{BRANCH}/2)^2*L_{BRANCH}$ (Supplementary Equation 3). The total volume occupied by BV was calculated as V_{BV} by summing up the volume of all branches. The imaged volume ($V_{IMAGING}$) was obtained by subtracting the volume occupied by peripheral inactive camera pixels ($_{DARK\ PIXELS}$) from the volume of imaged Z-stacks ($_{STACK}$) as follows: $V_{IMAGING} = (Height * Width * Depth)_{STACK} - (Height * Width * Depth)_{DARK\ PIXELS}$ (Supplementary Equation 4). The percentage of the volume occupied by BV in the upper dermis was calculated as $\%BV_{vol} = V_{BV} / V_{IMAGING} * 100$ (Supplementary Equation 5). Graph on the left shows the quantifications from four independent fields of view; image on the right represents the BV outline (in black) from one representative recorded field.

Supplementary Note 3. Extra analysis for certain trajectories-related quantities

The actual trajectory shapes can change considerably for the different individuals (Fig. 1a). Likewise, the MSD, if not taken in time windows (refer to the Hurst exponent section in the Methods section) tends to present strong variability, complicating statistical analysis. As illustrative examples, we show in the Supplementary Figure 4, the total traveled distance, i.e. the path length, as function of time for two specific parasites, one INV and one NINV. For these tracks we also display the MSD versus time (always calculated in relation to the starting point at $t=0$). Observe the distinct length scales in these two cases. Here, the INV parasite finally invade BV at the instant $t=804$ s. As discussed in the main text, for the NINV population, the k-means clustering of the MSD slopes (m) separate the Hurst exponents into three groups, c1, c2 and c3 (Fig. 3b). Such separation can be further understood from the Supplementary Fig. 4e, where we clearly see the overall trends, $m > 1$ for $H>1/2$ (super-diffusion), $m=1$ for $H=0.5$ (normal diffusion) and $m<1$ for $H<0.5$ (sub-diffusion).

As discussed in the main text, the Hidden Markov Model (HMM) identifies along the distinct tracks the trajectory stretches corresponding either to the St1 (low-motility) or to the St2 (high-motility) movement modes. A relevant information is thus the distribution of both size and duration of these St1 and St2 stretches modes, providing a general overview on the spatial-temporal ranges for the exploration/exploitation behaviors. This is shown in Supplementary Figure 5, which includes all the parasite tracks.

Finally, considering the entire collection of parasite tracks and four distinct time windows τ, we have obtained the corresponding MSD along each one of the stretches classified as St2 mode via the HMM. Then, we have generated the distributions of MSD lengths shown in the Supplementary Figure 6. The same procedure for the St1 has led to the distributions in the Supplementary Figure 7. We clearly see that the length sizes are much longer for the St2 mode. Moreover, in this case the distributions are much broader, presenting fatter tails.

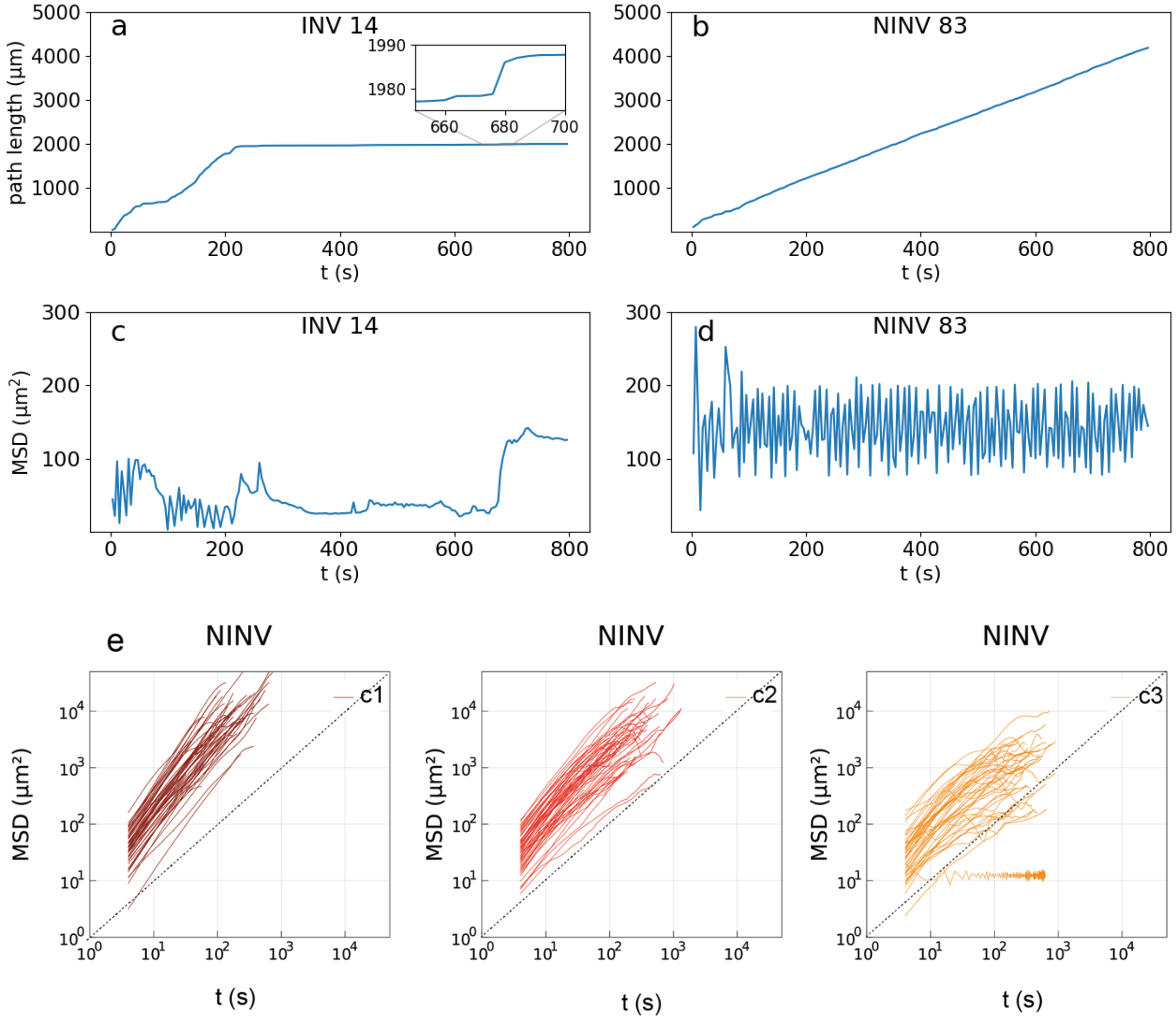

Supplementary Figure 4. Examples of traveling distances and Mean Square Displacement from the origin. (a-b) The path length, i.e. the total traveled distance, as function of time for two distinct parasites, one invader (INV) and one non-invader (NINV). **(c-d)** For these same trajectories, the Mean Square Displacement (MSD) versus time computed in reference to the tracks starting points at t=0. **(e)** MSD of NINV versus time in log-log scale corresponding to the data depicted in figure 3b of the main text. Here, the distinct MSD curves are clustered (c1: brown, c2: red and c3: orange) according to the kmeans protocol, selected by their slope values (m).

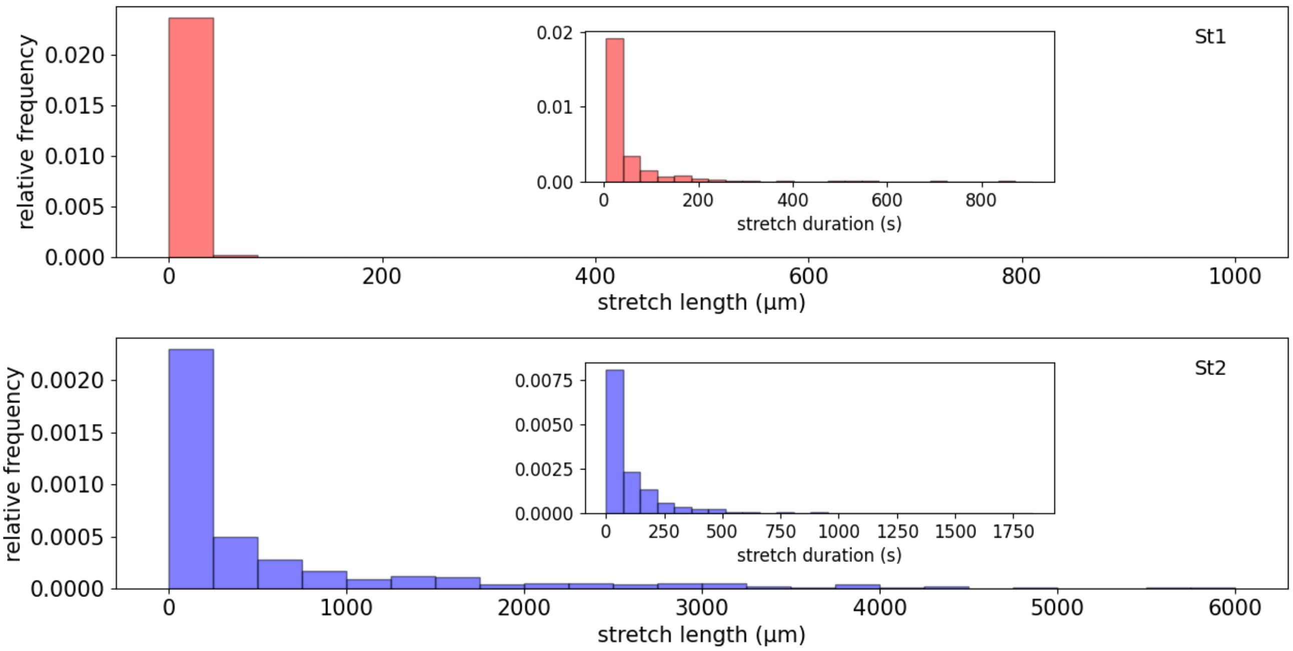

Supplementary Figure 5. Spatial and temporal distribution of the state 1 and state 2 movement modes. Distribution of the stretch lengths (in the inset, stretch times) for the state 1 (St1, red) and state 2 (St2, blue) locomotion modes as identified by Hidden Markov Model analysis. Here the full set of tracks for all parasites are considered (7 independent experiments, 188 tracks). As expected, the stretch lengths tend to be much longer for the high-motility St2 movement behavior.

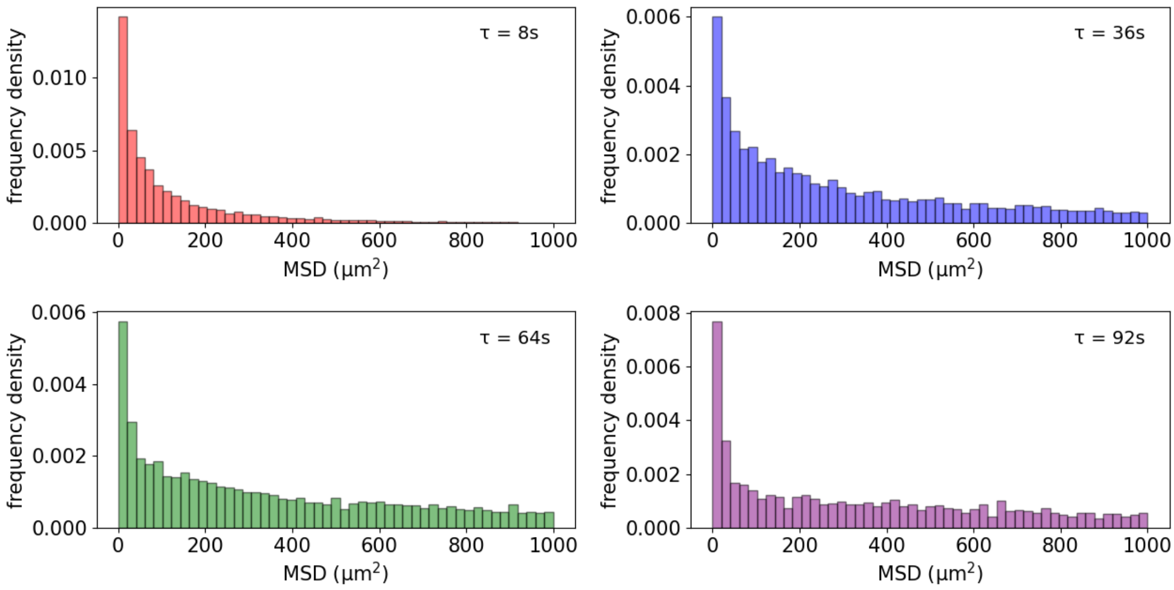

Supplementary Figure 6. Distribution of the Mean Square Displacement in different time windows of analysis for the state 2 mode. For time window values τ = 8 s (red), τ = 36 s (blue), τ = 64 s (green) and τ = 92 s (purple), the corresponding Mean Square Displacement (MSD) distributions resulting from all the stretches of the state 2 mode identified by the Hidden Markov Model analysis were computed for the entire set of parasites tracks (7 independent experiments, 188 tracks).

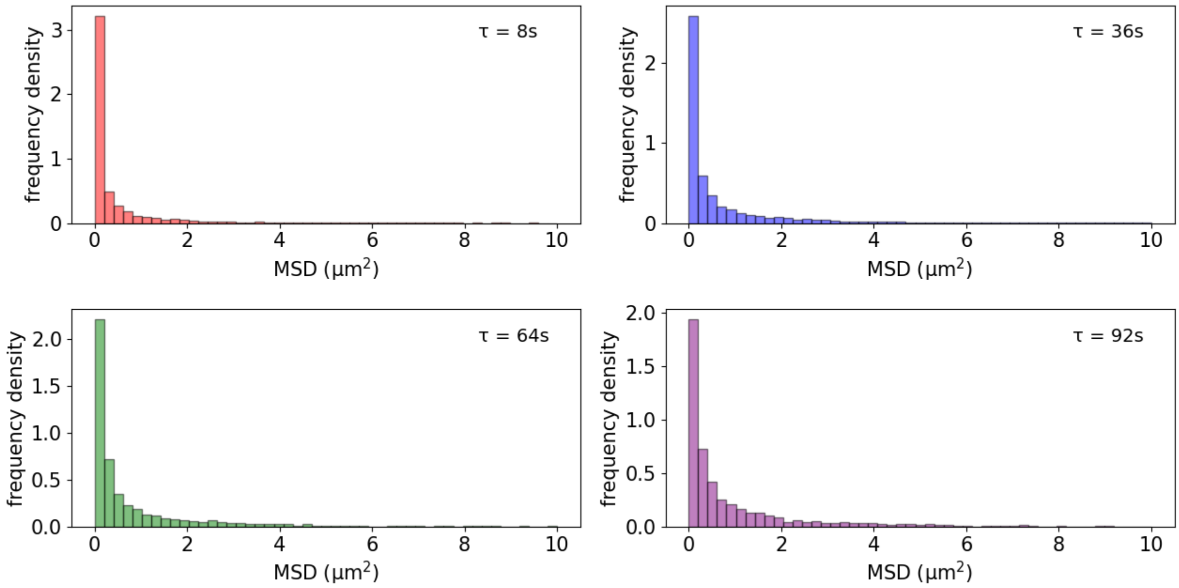

Supplementary Figure 7. Distribution of the Mean Square Displacement in different time windows of analysis for the state 1 mode. For time window values τ = 8 s (red), τ = 36 s (blue), τ = 64 s (green) and τ = 92 s (purple), the corresponding Mean Square Displacement (MSD) distributions resulting from all the stretches of the state 1 mode identified by the Hidden Markov Model analysis were computed for the entire set of parasites tracks (7 independent experiments, 188 tracks).

Supplementary References:

1. Box GEP, Jenkins GM, Reinsel GC, Ljung GM. *Time series analysis : forecasting and control*, Fifth edition / edn. John Wiley & Sons, Inc. (2016).

2. Viswanathan GM, da Luz MGE, Raposo EP, Stanley HE. *The Physics of Foraging: An Introduction to Random Searches and Biological Encounters*. Cambridge University Press (2011).

3. Méndez V, Campos D, Bartumeus F. Biological Searches and Random Animal Motility. In: *Stochastic Foundations in Movement Ecology: Anomalous Diffusion, Front Propagation and Random Searches* (eds Méndez V, Campos D, Bartumeus F). Springer Berlin Heidelberg (2014).

4. Shlesinger MF, Klafter J, Wong YM. Random-Walks with Infinite Spatial and Temporal Moments. *J Stat Phys* **27**, 499-512 (1982).

5. Klafter J, Blumen A, Shlesinger MF. Stochastic Pathway to Anomalous Diffusion. *Phys Rev A* **35**, 3081-3085 (1987).

6. Metzler R, Klafter J. The random walk's guide to anomalous diffusion: a fractional dynamics approach. *Phys Rep* **339**, 1-77 (2000).

7. Zaburdaev V, Denisov S, Klafter J. Levy walks. *Rev Mod Phys* **87**, 483-530 (2015).

8. Mantegna RN, Stanley HE. Stochastic-Process with Ultraslow Convergence to a Gaussian - the Truncated Levy Flight. *Physical Review Letters* **73**, 2946-2949 (1994).

9. Calenge C. The package “adehabitat” for the R software: A tool for the analysis of space and habitat use by animals. *Ecological Modelling* **197**, 516-519 (2006).
