# Supplementary material for: Plasmodium sporozoite search strategy to locate hotspots of blood vessel invasion
Source: Nat Commun. 2023 May 23;14:2965. doi: 10.1038/s41467-023-38706-z (PMC10205706; doi:10.1038/s41467-023-38706-z)
Supplement: Supplementary file 3 — Description of Additional Supplementary Information [file 41467_2023_38706_MOESM3_ESM.pdf]

Description of Additional Supplementary Information

Title: Supplementary Movie 1

Description: Sporozoites exhibit two types of motility regardless of their invasive phenotype. Example of an invader (cyan) and a non-invader (red) sporozoite switching from high-motility to low-motility mode upon contact with the same blood vessel. After failing to enter the bloodstream, the non-invader returns to the high-motility mode and eventually invades a lymph vessel. Scale bar: 10 µm.

Title: Supplementary Movie 2

Description: Sites of sporozoite intravasation into the microvasculature are clustered at hotspots delineated by the presence of pericytes. Example of two events of blood vessel invasion occurring in the vicinity of a pericyte. GFP expressing sporozoites and endothelial cells appear in green, CD31 and CD146 labelled structures are shown in magenta and white respectively. Note the GFP and CD31 negative, and CD146 positive body of the pericyte in white. Scale bar: 10 µm.
