# Supplementary material for: Plasmodium sporozoite search strategy to locate hotspots of blood vessel invasion
Source: Nat Commun. 2023 May 23;14:2965. doi: 10.1038/s41467-023-38706-z (PMC10205706; doi:10.1038/s41467-023-38706-z)
Supplement: Supplementary file 6 — Reporting Summary [file 41467_2023_38706_MOESM6_ESM.pdf]

nature portfolio

Corresponding author(s): Rogerio Amino
Marcos G.E. da Luz

Last updated by author(s): Apr 12, 2023

Reporting Summary

Nature Portfolio wishes to improve the reproducibility of the work that we publish. This form provides structure for consistency and transparency in reporting. For further information on Nature Portfolio policies, see our Editorial Policies and the Editorial Policy Checklist.

Statistics

For all statistical analyses, confirm that the following items are present in the figure legend, table legend, main text, or Methods section.

n/a	Confirmed	
☐	☒	The exact sample size (*n*) for each experimental group/condition, given as a discrete number and unit of measurement
☐	☒	A statement on whether measurements were taken from distinct samples or whether the same sample was measured repeatedly
☐	☒	The statistical test(s) used AND whether they are one- or two-sided<br>*Only common tests should be described solely by name; describe more complex techniques in the Methods section.*
☒	☐	A description of all covariates tested
☐	☒	A description of any assumptions or corrections, such as tests of normality and adjustment for multiple comparisons
☐	☒	A full description of the statistical parameters including central tendency (e.g. means) or other basic estimates (e.g. regression coefficient) AND variation (e.g. standard deviation) or associated estimates of uncertainty (e.g. confidence intervals)
☐	☒	For null hypothesis testing, the test statistic (e.g. *F*, *t*, *r*) with confidence intervals, effect sizes, degrees of freedom and *P* value noted<br>*Give P values as exact values whenever suitable.*
☒	☐	For Bayesian analysis, information on the choice of priors and Markov chain Monte Carlo settings
☒	☐	For hierarchical and complex designs, identification of the appropriate level for tests and full reporting of outcomes
☒	☐	Estimates of effect sizes (e.g. Cohen's *d*, Pearson's *r*), indicating how they were calculated

Our web collection on statistics for biologists contains articles on many of the points above.

Software and code

Policy information about availability of computer code

Data collection	All intravital imaging data were acquired using the Volocity software (PerkinElmer, version 6.4.0).

Data analysis

Image files were processed and analyzed using the Fiji software (Schneider et al., Nat Methods, 2012, version 2.0.0 to 2.9.0).Parasite tracks were determined manually using the MTrackJ plugin developed by Erik Meijering (Meijering et al., Methods in Enzymology, 2012, version 1.5.1). Analysis of the topological distribution of invasion events was performed using custom macros for Fiji to retrieve positions and determine projections or distances between neighbouring objects previously identified manually. The Lines8 plugin (version 2.13) designed by Gabriel Landini was used to determine the length of vascular segments separating projected positions of defined objects (entry site, branching point or centroid of pericyte bodies).

Hidden Markov Model (HMM) analysis of parasite tracks and power-law fits of the step length distributions were respectively performed with the moveHHM (version 1.0.1) and poweRlaw (version 0.70.6) packages from the R software (version 4.2.2).

Hurst exponents and k-means clustering were determined using custom algorithms in Python or in the Julia programming language.

Statiscal analyses were performed with the Prism software (GraphPad, version 9.5.1). Graphs were generated with either GraphPad Prism (version 9.5.1) or the R software (version 4.2.2). Illustrative examples from the supplementary figure 2 were generated with the R adehabitatHR package (version 0.4.20). Cartoons were created with BioRender.com.

All the codes used for the analysis of parasite motility are available online in a public GitHub repository under https://doi.org/10.5281/zenodo.7858654.

For manuscripts utilizing custom algorithms or software that are central to the research but not yet described in published literature, software must be made available to editors and reviewers. We strongly encourage code deposition in a community repository (e.g. GitHub). See the Nature Portfolio guidelines for submitting code & software for further information.

Data

Policy information about availability of data

All manuscripts must include a data availability statement. This statement should provide the following information, where applicable:

- Accession codes, unique identifiers, or web links for publicly available datasets
- A description of any restrictions on data availability
- For clinical datasets or third party data, please ensure that the statement adheres to our policy

Raw parasite track data generated in this study have been deposited in a public GitHub repository accessible under https://doi.org/10.5281/zenodo.7858654. The processed data resulting from the analysis of intravital movies and parasite tracks are provided with this paper in the Source Data file.

Human research participants

Policy information about studies involving human research participants and Sex and Gender in Research.

Reporting on sex and gender	N/A
Population characteristics	N/A
Recruitment	N/A
Ethics oversight	N/A

Note that full information on the approval of the study protocol must also be provided in the manuscript.

Field-specific reporting

Please select the one below that is the best fit for your research. If you are not sure, read the appropriate sections before making your selection.

[x] Life sciences   [ ] Behavioural & social sciences   [ ] Ecological, evolutionary & environmental sciences

For a reference copy of the document with all sections, see nature.com/documents/nr-reporting-summary-flat.pdf

Life sciences study design

All studies must disclose on these points even when the disclosure is negative.

Sample size

No statistical methods were used to predetermine sample size. Instead, data were pooled from independent experiments to cumulate >100 sporozoite tracks with >15% sampling of the total parasite population for sporozoite behavior analysis (Fig 1-3) or > 50 invasion sites to analyze their topological distribution, taking into account heterogeneities between recorded fields. Such sample sizes are in line with prior studies from the same field of research (e.g. Hopp et al., eLife, 2015).

Data exclusions

Intravital imaging experiments during which none or only one single event of blood vessel invasion was recorded were not included in the

	analysis. For the analysis of sporozoite motility and search strategy, only parasites that could be tracked unequivocally for at least 60 s for invaders and 90 s for non-invaders were included, which enabled sampling of 17% to 48% of the population of sporozoites initially present in the recorded field of view. For the analysis of step length distributions, data points associated with mixed motility modes were not included.
Replication	One independent experiment refers to a 40-minute long longitudinal recording of freshly dissected salivary gland sporozoites micro-injected into one mouse ear. All intravital imaging experiments presented in the manuscript were repeated at least 4 times in independent animals and using sporozoites from at least 4 independent mosquito infections. Reported findings were replicated across all replicates with similar trends and data were pooled for statistical analysis. Specifically, analysis of sporozoite motility and search strategy (Fig. 1-3) was performed on tracks of 108-188 sporozoites collected over 4-7 independent experiments (4-6 mice, 6 mosquito infections). The clusterization of invasion sites was evidenced with 5 independent experiments (5 mice, 5 mosquito infections) analyzing a total of 64 invasion events. The topological distribution of invasion sites in respect with branching points and pericytes was determined analyzing respectively 91 and 74 invasion events, recorded over 13 independent experiments each (13 mice, 8 mosquito infections). Pericyte distribution along the microvasculature was assessed over 10 independent field of views, analyzing 47 cells.
Randomization	Randomization was not relevant for this study. Intravital imaging experiments involved a single animal at a time and analysis of sporozoite motility and invasive phenotype was restricted to a single combination of wild-type-like parasites within wild-type-like rodent hosts.
Blinding	Blinding was not relevant for this study since analysis of sporozoite motility and invasive phenotype was restricted to a single combination of wild-type-like parasites within wild-type-like rodent hosts. Determination of motility states, step length distributions, Hurst exponents as well as topological characterization of invasion sites relied on the use of computing algorithm, thus limiting human error and bias.

Reporting for specific materials, systems and methods

We require information from authors about some types of materials, experimental systems and methods used in many studies. Here, indicate whether each material, system or method listed is relevant to your study. If you are not sure if a list item applies to your research, read the appropriate section before selecting a response.

Materials & experimental systems

n/a	Involved in the study
☐	☒ Antibodies
☒	☐ Eukaryotic cell lines
☒	☐ Palaeontology and archaeology
☐	☒ Animals and other organisms
☒	☐ Clinical data
☒	☐ Dual use research of concern

Methods

n/a	Involved in the study
☒	☐ ChIP-seq
☒	☐ Flow cytometry
☒	☐ MRI-based neuroimaging

Antibodies

Antibodies used	1. Alexa Fluor 647 anti-mouse CD31 antibody (Biolegend, clone MEC13.3, Cat. #102516, Lot B139130)<br>2. Alexa Fluor 488 anti-mouse CD31 antibody (Biolegend, clone 390, Cat. #102414, Lot B239624)<br>3. Alexa Fluor 488 anti-mouse CD146 antibody (Biolegend, clone ME-9F1, Cat. #134707, Lot B140756)<br>4. PE anti-mouse CD146 antibody (Biolegend, clone ME-9F1, Cat. #134703, Lot B131672)<br>5. Alexa Fluor 647 anti-mouse CD146 antibody (Biolegend, clone ME-9F1, Cat.#134718, Lot B247474)
Validation	All antibodies used in this study are commercially available and have been validated by the manufacturer:<br>1. https://d1spbj2x7qk4bg.cloudfront.net/en-us/products/alexa-fluor-647-anti-mouse-cd31-antibody-3094?pdf=true&displayInline=true&leftRightMargin=15&topBottomMargin=15&filename=Alexa%20Fluor%C2%AE%20647%20anti-mouse%20CD31%20Antibody.pdf&v=20220429052301<br>2. https://d1spbj2x7qk4bg.cloudfront.net/en-us/products/alexa-fluor-488-anti-mouse-cd31-antibody-3091?pdf=true&displayInline=true&leftRightMargin=15&topBottomMargin=15&filename=Alexa%20Fluor%C2%AE%20488%20anti-mouse%20CD31%20Antibody.pdf&v=20220831123135<br>3. https://d1spbj2x7qk4bg.cloudfront.net/en-us/products/alexa-fluor-488-anti-mouse-cd146-antibody-6864?pdf=true&displayInline=true&leftRightMargin=15&topBottomMargin=15&filename=Alexa%20Fluor%C2%AE%20488%20anti-mouse%20CD146%20Antibody.pdf&v=20220831123135<br>4. https://d1spbj2x7qk4bg.cloudfront.net/en-us/products/pe-anti-mouse-cd146-antibody-6027?pdf=true&displayInline=true&leftRightMargin=15&topBottomMargin=15&filename=PE%20anti-mouse%20CD146%20Antibody.pdf&v=20220614123010<br>5. https://d1spbj2x7qk4bg.cloudfront.net/en-us/products/alexa-fluor-647-anti-mouse-cd146-antibody-15112?pdf=true&displayInline=true&leftRightMargin=15&topBottomMargin=15&filename=Alexa%20Fluor%C2%AE%20647%20anti-mouse%20CD146%20Antibody.pdf&v=20220614123010

Animals and other research organisms

Policy information about studies involving animals; ARRIVE guidelines recommended for reporting animal research, and Sex and Gender in Research

Laboratory animals	Female Anopheles stephensi mosquitoes (SDA 500 strain) were reared in the Center for Production and Infection of Anopheles at the Institut Pasteur and infected 3-4 days after emergence. Following parasite transmission, mosquitoes were kept in a climatic chamber at 21°C, 80% humidity under a 12-hour dark/light cycle with sucrose at their disposition and were fed on naive mice 7 days after the infectious blood meal.<br><br>Four to six week-old female RjOrl:Swiss mice purchased from Janvier Labs were used to provide infectious and non-infectious blood meals to mosquitoes. Intravital imaging experiments were performed on 5-10 week-old C57BL/6JRj female mice purchased from Janvier Labs or flk1-GFP female mice (Xu et al., J Cell Biol, 2010) bred at the Institut Pasteur on a C57BL/6 background. Animals were housed in individually ventilated cages in the animal facilities of the Institut Pasteur accredited by the French Ministry of Agriculture. Mice were kept under standard conditions (10/14 hour dark/light cycle, temperature 20°C +/- 2°C, humidity 50% +/- 10%) with irradiated rodent feed and autoclaved water ad libitum.
Wild animals	The study did not involve wild animals.
Reporting on sex	This study focuses on the behaviour of Plasmodium berghei sporozoites, which are asexual stages of the parasites and intravital imaging was performed in the skin of female mice. A study investigating the influence of sex on the susceptibility of animals to an intradermal P. berghei sporozoite challenge did not highlight any significant differences between naive males and females (Vom Steeg et al., Vaccine, 2019), suggesting host sex does not have a major influence on parasite behavior in the skin.
Field-collected samples	The study did not involve samples collected from the field.
Ethics oversight	All animal experiments were approved by the Ethics Committee #89 (CETEA, Institut Pasteur) and registered under the reference 01324.02, 32422 and 32989 by the French Ministry of Higher Education, Research and Innovation.

Note that full information on the approval of the study protocol must also be provided in the manuscript.
